# Blood inflammatory and endothelial markers in women with von Willebrand disease

**DOI:** 10.1371/journal.pone.0210544

**Published:** 2019-01-10

**Authors:** Igor Govorov, Katarina Bremme, Anders Larsson, Margareta Holmström, Eduard Komlichenko, Roza Chaireti, Miriam Mints

**Affiliations:** 1 Department of Women’s and Children’s Health, Karolinska Institutet, Stockholm, Sweden; 2 Department of Medical Sciences, Uppsala University, Uppsala, Sweden; 3 Department of Medicine, Karolinska University Hospital Solna, Stockholm, Sweden; 4 Institution of Pediatrics and Perinatology, Almazov National Medical Research Centre, Saint-Petersburg, Russia; 5 Department of Molecular Medicine and Surgery, Karolinska Institutet, Stockholm, Sweden; 6 Department of Hematology, Karolinska University Hospital, Stockholm, Sweden; Institut d'Investigacions Biomediques de Barcelona, SPAIN

## Abstract

**Introduction:**

VWD-affected females often experience menorrhagia. Periodical fluctuations of the sex steroids during the menstrual cycle cause changes both in the coagulation and immune system. The aim of the current study was to assess the changes in selected inflammatory and endothelial markers in women with VWD during two phases of the menstrual cycle (follicular and luteal) and to compare it with corresponding data from healthy controls.

**Materials and methods:**

The study group included 12 VWD-affected females with regular menstrual cycle, with none of them being prescribed hormone treatment. They were not pregnant or breastfeeding. The control group consisted of 102 healthy females, matched for age and BMI.

**Results:**

Within the VWD group, endostatin was higher during the follicular phase, compared to the luteal phase, although the difference was not significant (p = 0.062). sICAM-1 and IL-6 were higher in VWD-affected females, compared to the controls, sVCAM-1, cathepsin S and sP-selectin were lower (p<0.003 for all cases). The pattern was constant throughout the menstrual cycle.

**Conclusions:**

Higher levels of endostatin during early follicular phase could potentially predispose women with VWD to the development of heavy menstrual bleeding, due to antiangiogenic properties and ability to suppress several coagulation factors. Lower p-selectin levels in VWD group, compared to controls, may also contribute to the bleeding tendency. Changes in other proteins, involved in angiogenesis are hypothetically related to the formation of angiodysplasia—common complication of VWF deficiency. The latter statement requires confirmation in larger studies.

## Introduction

Von Willebrand disease (VWD) is a blood-clotting dysfunction with a prevalence reaching 1% of the general population, making it the most common inherited bleeding disorder worldwide [1]. It results from a deficiency in quantity/quality of the specific blood glycoprotein—von Willebrand factor (VWF), which ultimately leads to the prolonged bleedings. Women, affected by VWD frequently suffer from excessive bleedings during menstruation.

In addition to a major role both in primary and secondary hemostasis, VWF participates in endothelial functioning and angiogenesis [2]. Moreover, VWF levels rise during inflammation and VWF is involved in acute phase reactions [3–5].

Cyclic change in sex steroids levels during the menstrual cycle is one of the characteristic features of the female body. At the same time, the effects caused by these fluctuations extend far beyond the boundaries of the reproductive system. These effects are so pronounced that researchers even speculate on whether female health is generally cyclical [6].

Several inflammatory mediators have been reported to cyclically fluctuate during the menstrual cycle.

C-reactive protein (CRP) is a member of the pentraxin superfamily with the liver being the primary production site. Remaining most of the time at low or undetectable level, CRP increases thousandfold during inflammation, whether infectious or sterile, with interleukin-6 (IL-6) as a major stimulus. Several studies concluded that estrogen decreases, while progesterone increases CRP [7–9].

IL-6 is a well-known multifunctional cytokine, participating in different processes, both pro- and anti-inflammatory. IL-6 was found to be highest preovulatory and lowest during the luteal phase, which possibly implies its dependence on the progesterone levels [10]. However, other studies reported no sustained cyclic variations in IL-6 levels throughout the menstrual cycle [11, 12].

Cell adhesion molecules is a large group of proteins involved in binding cells together or to extracellular matrix. The intercellular adhesion molecule 1 (ICAM-1), vascular cell adhesion molecule 1 (VCAM-1), E-selectin, P-selectin are all included in this group. ICAM-1 and VCAM-1 were shown to be at a higher concentration during the follicular phase with further decline during the luteal phase [13, 14]. E-selectin and P-selectin are considered to be dependent on the concentration of sex hormones, although there is lack of studies and the results are conflicting [15, 16].

Cathepsins form a family of proteases with versatile structure and catalytic activity predominantly operating within the lysosomes. Several cathepsins reside both in eutopic endometrium and endometriotic lesions [17, 18], where they could activate matrix metalloproteinases or urokinase-type plasminogen activator [19–21]. At least some of the cathepsins are dependent on sex hormones levels, albeit it was only demonstrated in ewes [22].

Endostatin is formed from collagen type XVIII during proteolytic cleavage by cathepsin L [23]. It functions mainly as endogenous inhibitor of angiogenesis, but has been also reported to modulate coagulation cascade, through stimulating plasmin production and downregulating VWF, tissue factor, FVII, FX, FXI and FXII [24, 25].

Moreover, sex hormones are well-known modifiers of such important processes responsible for organism survival, such as blood clotting and immunity. Coagulation and inflammatory systems are in their turn intimately intertwined, not only influencing each other but also sharing common pathways and effectors [26, 27]. Therefore, it is interesting what happens with this finely tuned interaction, in the presence of a known defect in the hemostasis, for example, in VWD.

The aim of the current study was to analyze changes in the blood levels of some inflammatory and endothelial markers during menstrual cycle in women with von Willebrand disease and thereby try to elucidate interactions between the inflammatory and hemostatic systems.

## Materials and methods

This was an observational study, measuring inflammatory and endothelial markers on the same group of women during two phases of menstrual cycle.

The inclusion criteria were as follows: Female patients with von Willebrand disease, age 18–52 (pre-menopausal), with regular menstrual cycle (21–35 days), none of them being prescribed hormone medications (combined oral contraceptives, contraceptive implants, intrauterine devices, HRT) or anti-inflammatory drugs (NSAIDs) at the time of blood sampling. Pregnant and breast-feeding women were excluded, as were those who either had irregular menstrual cycle or even none, due to physiological or iatrogenic menopause. In total 12 patients were included. Blood samples were collected twice for each patient: one during follicular phase (cd 2–5) and one during luteal phase (cd 22–25). The blood levels of the following components were assessed: interleukin-6 (IL-6), endostatin, high sensitivity C-reactive protein (hs-CRP), soluble E-selectin and P-selectin (sE-selectin and sP-selectin), intracellular and vascular cell adhesion molecules (ICAM-1 and VCAM-1) and cathepsins L and S. The results were then compared with those from healthy controls (n = 102), coming from the recent study by Chaireti et al. [28].

In all patients venous blood samples were drawn from an antecubital vein after 15 min in the supine position. All samples were drawn in the morning after an overnight fast. Blood samples for analysis of coagulation factors were collected in citrated tubes and immediately centrifuged at 2000g for 15 min. After removal of the cells, plasma was re-centrifuged for another 15 min at 2000g. Cell-free plasma was stored at -70°C until analyzed.

The current study is done in cooperation with Almazov National Medical Research Centre, Saint-Petersburg, Russia.

### Ethical permission

All participants were informed of the study. Written informed consent was obtained from all participants. Personal data was made anonymous directly after collection. The current study was approved by Stockholm Regional Ethics Committee (№ 2016/503-31) and was subsequently supplemented with the local permission from Almazov National Medical Research Centre in Saint-Petersburg, Russia (№ 17/ПЦ).

### Measurement of inflammatory and endothelial markers

High sensitivity C-reactive protein was analyzed on a BS380 instrument (Mindray, Shenzhen, China) with CRP reagents (CRP-6K26) from Abbott Laboratories (Abbott Park, IL, US). The total coefficient of variation (CV) for the CRP method was 6.9% at 1.30 mg/L.

Cathepsin L (DY952), Cathepsin S (DY1183), Endostatin (DY1098), sE-selectin (DY724), ICAM-1 (DY720), IL6 (DY206), sP-selectin (DY137), and VCAM-1 (DY809) were analyzed by the means of commercially available ELISA kit (R&D Systems, Minneapolis, MN, USA) according to the instructions of the manufacturer. The total CVs of the ELISAs were approximately 7%.

### Statistical analysis

Statistical analysis was performed using SPSS 24 for Mac OS. Background data between the groups was compared using unpaired two-sample t-test.

Since there are no studies with a design similar to ours, *i*.*e*. measuring inflammatory markers in VWD patients during the menstrual cycle, we calculated the required power for the cohort by using results from the studies by Chaireti *et al*. [29] and Rugeri *et al*. [30], where thrombin generation was measured in healthy women and patients with von Willebrand disease, respectively. The required cohort size in order to achieve a power of 0.8 with a type I-error of 5% was 12 patients.

Non-parametric tests were chosen for statistical calculations since the number of patients was small and the data was not normally distributed. We used the Wilcoxon signed-rank test to compare changes in inflammatory and endothelial variables during menstrual cycle within study group. Mann-Whitney U test was employed to assess differences between control and study groups. In all cases an exact p-value was calculated, while statistical significance was set at p < 0,05.

## Results

The study group included 12 female patients with VWD. Their age was 35.0 (33.0–41.0) (hereinafter [Median (25–75 percentiles)] and BMI = 23.1 (20.2–29.4). VWD types distribution was as follows: type 1–7 patients, type 2–3 patients (with 2 of them having subtype 2M), unspecified—2 patients. The raw dataset is deposited to a public repository:

https://doi.org/10.6084/m9.figshare.7422620.v1

We started with comparing inflammatory and endothelial markers within the study group during follicular (cd 2–5) and luteal phases (cd 22–25) of the menstrual cycle. The results are presented as median (25–75 percentiles) in Table 1. None of the parameters differed between phases, with the most pronounced differences found in endostatin concentrations (p = 0.062).

**Table 1:** Inflammatory and endothelial markers during follicular and luteal phase of menstrual cycle in patients with VWD.

	Follicular phase (cd 2–5)	Luteal phase (cd 22–25)	p-value
CATHL, pg/mL	6475.05 (3363.98–16656.98)	3444.47 (2091.28–16175.62)	0.875
CATHS, pg/mL	4137.74 (2950.11–5524.64)	4686.90 (3743.88–6143.64)	0.239
Endostatin, pg/mL	98066.45 (55628.48–132801.84)	71986.73 (52809.96–106878.43)	0.062
hs-CRP, mg/L	1.34 (0.38–2.23)	0.47 (0.27–1.41)	0.155
IL-6, pg/mL	3.99 (2.39–12.41)	3.64 (1.11–8.88)	0.508
sE-selectin, pg/mL	20686.28 (16741.31–31344.94)	23751.68 (15575.59–30806.03)	0.433
sICAM-1, pg/mL	193119.66 (177125.86–217278.64)	173952.76 (140408.86–196667.53)	0.209
sP-selectin, pg/mL	17945.70 (12700.38–27885.64)	19067.49 (13170.77–29360.94)	0.638
sVCAM-1, pg/mL	278598.80 (259183.24–328763.73)	276624.05 (248947.58–303147.40)	0.754

CATHL, cathepsin L; CATHS, cathepsin S; hs-CRP, high sensitivity C reactive protein; IL-6, interleukin 6; sE-selectin, soluble E-selectin; sP-selectin, soluble P-selectin; sICAM-1, soluble intracellular cell adhesion molecule 1; sVCAM-1, soluble vascular cell adhesion molecule 1

**Fig 1:**
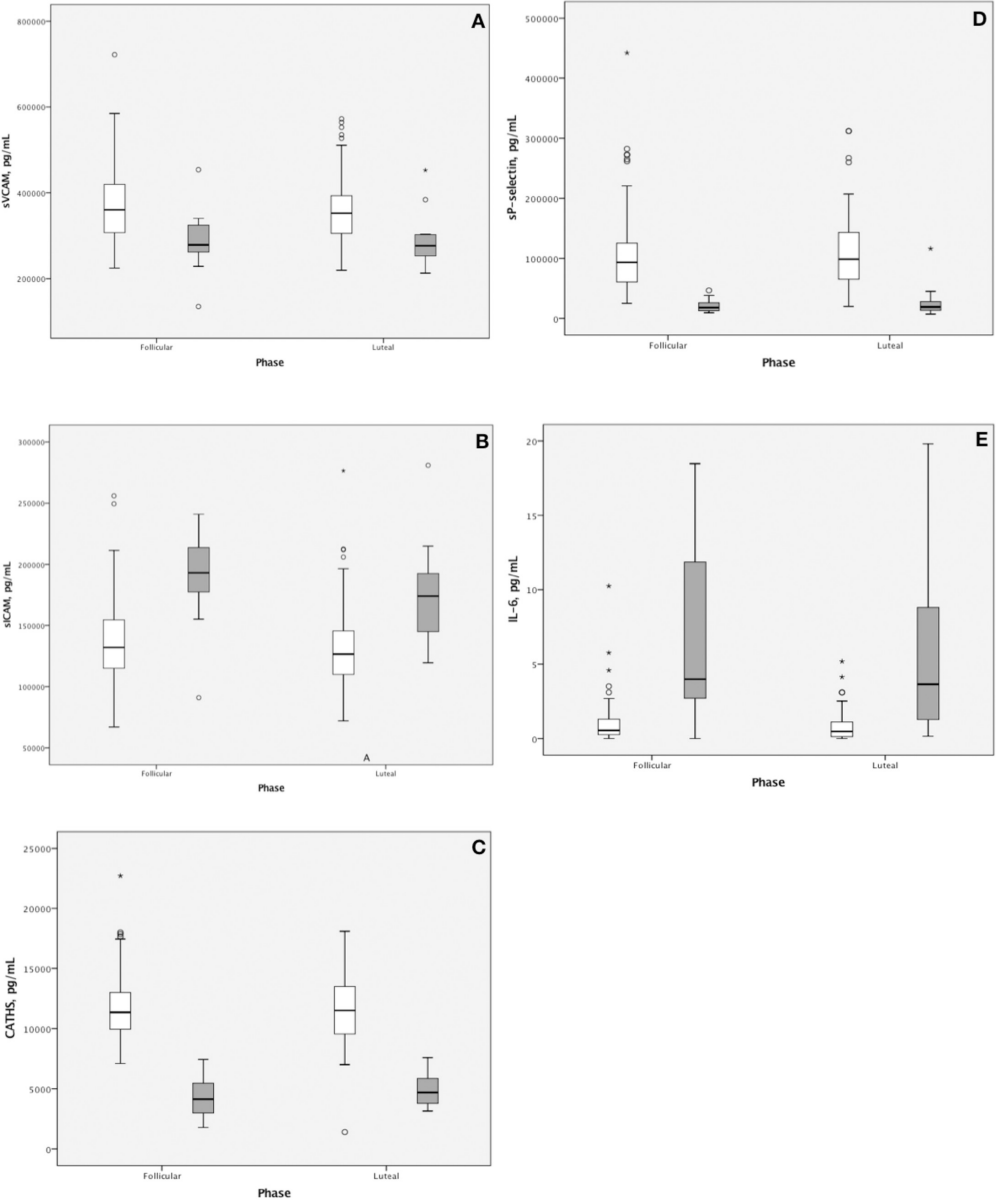
Comparison of those inflammatory and endothelial markers that differ significantly between the groups. VWD patients are marked in grey, while healthy controls in white. In all cases p-value was <0.0005, except for sVCAM-1 (Fig 1A), where it was p = 0.002 and p = 0.001 during follicular and luteal phase respectively. CATHS, cathepsin S; IL-6, interleukin 6; sICAM-1, soluble intracellular adhesion molecule 1; sVCAM-1, soluble vascular cell adhesion molecule 1; sP-selectin, soluble P-selectin.

In addition, we compared the levels of the inflammatory and endothelial markers between VWD patients and controls separately for each phase of the menstrual cycle. Several variables, to be concrete sICAM-1, sVCAM-1, cathepsin S, IL-6 and sP-selectin, differed significantly between the groups and this set was constant during both phases of the menstrual cycle. The boxplots are present in Fig 1.

## Discussion

In the current study we found higher levels of the endostatin during the follicular phase compared to the luteal phase in women with VWD. We observed higher sICAM-1 and IL-6 levels in VWD-affected females, compared to the healthy controls. On the contrary, sVCAM-1, cathepsin S and sP-selectin were lower in the study group. To the best of our knowledge, inflammatory markers during the menstrual cycle in women with VWD have not yet been studied.

Growing evidence suggests that periodic fluctuations of sex steroids, primarily estrogen and progesterone, throughout the menstrual cycle distinctly influence the functioning of the immune system. Briefly, one can state that the activity of the immune system is higher during the follicular phase, which is at least partly due to higher levels of the endogenous estrogen[6, 31]. Conversely, during the luteal phase the activity of the immune system decreases reflecting the rise in progesterone, which has anti-inflammatory properties [6, 32]. During menstruation, an acute inflammatory event, the immune system becomes active again [33, 34].

Endostatin tended to be higher during follicular phase in women with VWD, though the p-value was slightly above the established boundary(p = 0.062). It is known that the early follicular phase (cd 2–5) corresponds to the menstruation phase of the uterine cycle, during which active angiogenesis takes place in the basal layer of the endometrium. Therefore, it sounds reasonable to hypothesize that increase in antiangiogenic endostatin, which also downregulated VWF and several other coagulation factors, during early follicular phase may contribute to the development of menorrhagia—common symptom in women with VWD. However, taken into consideration that VWF also modulates angiogenesis, the above observation warrants further investigation [35].

In the current study on VWD-affected females this tendency was not that pronounced with the most measured variables not being significantly different between two phases of the menstrual cycle.

It is possible that the absence of the significant differences in variables throughout the menstrual cycle is caused by a small number of observations within the study group. At the same time, one should keep in mind that inflammatory system sensitively reacts to the changes in internal and external environment, for instance age, birth weight [36], BMI [37], smoking habits [38], physical activity [39] and possibly many other yet unknown factors. Therefore, it seems impossible to completely avoid the confounders.

Increase in circulating soluble adhesion molecules was reported for a range of conditions and usually is a sign of either endothelial activation or local/systemic inflammation [40, 41].

P-selectin is a protein stored together with VWF in Weibel-Palade bodies in endothelial cells and alpha-granules within thrombocytes. Upon stimulation with triggers, for instance thrombin, p-selectin expresses on the surface of endothelial cells and platelets, facilitating their aggregation during hemostasis. We found lower sP-selectin levels in women with VWD, compared with healthy females. This observation strengthens the idea about intimate interactions between p-selectin and VWF [42]. Furthermore, we hypothesize that decrease in sP-selectin may contribute to the bleeding manifestations in women with VWD.

In the current study sVCAM-1 levels were lower while sICAM-1 higher in women with VWD compared to healthy controls and this tendency persisted during both phases of the menstrual cycle. VCAM-1 is generously expressed on endothelial cells, facilitating the adhesion between endothelium and leukocytes. At the same time, VCAM was shown to participate in *de novo* vessel formation, including cytokines-induced neoangiogenesis [43, 44].

sICAM-1 induces transendothelial migration of leukocytes, which precedes neoangiogenesis within the inflammatory *loci* [45]. However, sICAM-1 fluctuates significantly due to various benign and malignant diseases, as well as lifestyle-factors, as described in the overview by Witkowska&Borawska [46]. Interestingly, increase in sICAM-1 is triggered with a range of cytokines, *i*.*a*. IL-6. The latter was also higher in women with VWD compared to healthy controls. IL-6 is a pleiotropic cytokine, which promotes a variety of predominantly proinflammatory processes, although ant-inflammatory properties of IL-6 are also reported. Among the different processes in which IL-6 is involved, it is also interesting to note its effect on angiogenesis. In the recent work by Gopinathan et al. [47] was demonstrated that IL-6 stimulate defective angiogenesis.

Cathepsin S was lower in women with VWD, compared to controls. Cathepsin S plays an essential role in antigen presentation, extracellular matrix degradation, the latter being critical for angiogenesis. It was demonstrated that deficiency in cathepsin S causes inhibition of angiogenesis [48, 49].

Therefore, we observe a dysregulation of pro- and anti-angiogenic mediators in women with VWD, which may be important with regard to the specific complication of VWD—formation of angiodysplastic lesions in different body regions [50, 51]. This condition is considered to arise from VWF-dependent negative modulation of angiogenesis through multiple pathways [35]. One must however state, that our study was not initially designed to study angiodysplasia in VWD patients, and therefore this founding needs additional verification.

It is well known, that women with VWD are at higher risk of experiencing excessive bleeding during menstruation. In the current study, we observed higher endostatin levels during the early follicular phase compared with the luteal phase in women with VWD. Taking into consideration that endostatin suppresses angiogenesis and decreases a range of procoagulant factors (including VWF), increase in endostatin may probably contribute to the development of heavy menstrual bleeding in this cohort. In addition, the development of the bleeding complications may be due to the decrease in p-selectin levels among women with VWD, compared to healthy controls. Furthermore we detected an imbalance of pro- and anti-angiogenic mediators in women with VWD, which maybe can contribute to the formation of the vascular ectasias—angiodysplasia—common founding in patients with VWD. However, the latter observation needs further approval in larger studies.
